# What's in the telling? Understanding social, psychological and clinical aspects of HIV disclosure

**DOI:** 10.1080/09540121.2015.1102687

**Published:** 2015-11-29

**Authors:** Xiaoming Li, Shan Qiao, John de Wit, Lorraine Sherr

**Affiliations:** ^a^ Department of Health Promotion, Education, and Behavior, South Carolina SmartState Center for Healthcare Quality (CHQ), University of South Carolina Arnold School of Public Health, Columbia, SC, USA; ^b^ Center for Social Research in Health, University of New South Wales, Sydney, New South Wales, Australia; ^c^ Research Department of Infection & Population Health, University College London, London, UK

The course of HIV infection includes a parallel pathway of disclosure (Asudani, Corser, & Patel, 2004). Multiple types of HIV disclosure have been identified across a wide spectrum of HIV prevention and treatment cascade (Arnold, 2008; Arun, Singh, Lodha, & Kabra, 2009; Ateka, 2006; Bairan et al., 2007). They all need to be under the spotlight of academic research. HIV testing, often seen as an entry point to diagnosis and disease management, involves disclosure of risk behaviors and disclosure of partners on the part of the individual being tested and disclosure of test results on the part of the healthcare providers (Atuyambe et al., 2014; Bachanas et al., 2013; Bedell, van Lettow, & Landes, 2014). Onward disclosure of serostatus is then the complex behavior under consideration in most HIV disclosure research (Afifi & Afili, 2009).

Disclosure, or self-disclosure, is a process in which personal (often private or confidential) information is verbally communicated from one person (i.e., the discloser) to another person (i.e., the target; Chelune, 1979). HIV disclosure includes an array of behaviors associated with the practice in which HIV-infected persons disclose their HIV serostatus to their partners, family members, or friends; or when a child is informed of her/his own HIV status (Qiao, Li, & Stanton, 2013b). Essentially, it is the process of moving from unawareness to knowledge, invariably a unidirectional, irrevocable act (Li, de Wit, Qiao, & Sherr, 2015). Disclosure has been viewed as an integral component in the public health effort to reduce incident HIV infections and improve HIV treatment and care (Remis, 2013). In detail, much research has also been done in terms of factors enabling or hindering disclosure, correlates and predictors of disclosure, preparation for disclosure and consequences of disclosure (Jorjoran Shushtari, Sajjadi, Forouzan, Salimi, & Dejman, 2014; Kumar, Waterman, Kumari, & Carter, 2006; Latkin et al., 2012; Lee, Bastos, Bertoni, Malta, & Kerrigan, 2014; Liamputtong & Haritavorn, 2014; Linda, 2013; Lyimo et al., 2014).

Existing research and theorizing suggest that HIV disclosure is not a single event but a process of gradual and selective delivery of information embedded in the context of a social relationship (Lesch et al., 2007; Moses & Tomlinson, 2013). A growing number of empirical studies have focused on the process of HIV disclosure, including decision-making, disclosure patterns, and post-disclosure adjustment. These elements are shaped by various social, psychological and clinical factors (Bott & Obermeyer, 2013; Letteney, Krauss, & Kaplan, 2012; Li et al., 2007). Studies have elaborated a cycle of how HIV disclosure has had complex, often reciprocal influences on behavioral, psychosocial, and clinical aspects of the lives of HIV-infected individuals (Butler et al., 2009; Carballo-Dieguez, Miner, Dolezal, Rosser, & Jacoby, 2006; Dageid, Govender, & Gordon, 2012; De Baets, Sifovo, Parsons, & Pazvakavambwa, 2008). Disclosure has also been found to be layered with different needs, demands, and ramifications of various forms of disclosure ranging from sexual partners, to close family members, to friends and social acquaintances, to children, to employers, and to employees (Eustace & Ilagan, 2010; Fesko, 2001). Conceptual frameworks need to provide understanding of the drivers and inhibitors of disclosure, on variations in the disclosure process and how these may (or may not) affect various physical or psychosocial outcomes (Chaudoir & Fisher, 2010; Chaudoir, Fisher, & Simoni, 2011; Qiao et al., 2013b). Both intended and unintended consequences of disclosure need to be considered (Serovich, McDowell, & Grafsky, 2008; Shamu, Zarowsky, Shefer, Temmerman, & Abrahams, 2014). Active, coerced, and accidental disclosure may all have different effects on the behavior and mental health of all individuals concerned (Feigin, Sapir, Patinkin, & Turner, 2013).

Over the course of the HIV, epidemic disclosure has been an area of concern, often with few parallels in other disease conditions. In the early days of the epidemic, pre- and post-test counseling cautioned about social reactions to disclosure and provided specific advice and guidance on the issue (CDC, 1987, 1993, 2001; WHO, 2011). Legal responses to non-disclosure have spawned an entire criminalization of HIV spread issue (Holmes & O'Byrne, 2006; Lichtenstein, Whetten, & Rubenstein, 2014; Stein et al., 1998). Psychological responses to disclosure have found direct adaptation, support, mental health, and resilience sequelae (Qiao, Li, & Stanton, 2013a; Smith, Rossetto, & Peterson, 2008). Social research has identified relationship implications from intimate partners to community members (Bairan et al., 2007). Research on age and disclosure has shown the diverse needs, requirements, and outcomes for various age cohorts (Chaudoir et al., 2011; Hawk, 2007). Most importantly, the public health response has clearly articulated the fundamental requirement of full disclosure if inroads to HIV prevention are to be made (Chaiyamahapurk, Pannarunothai, & Nopkesorn, 2011; Remis, 2013).

We are pleased to present in this special issue, a collection of papers on the social, psychological, and clinical aspects of HIV disclosure. This special issue was designed in conjunction with a special issue recently published in AIDS by the same guest editors (Li et al., 2015). These two compilations provide a gathering of insight into social, psychological, and clinical aspects of HIV disclosure within this collection, and an exploration of issues around disclosure intervention design and development in the other collection (Li et al., 2015).

The papers in this special issue address topics related to different types of HIV disclosure, reflecting various social relationships between the disclosers and the potential targets of the disclosure, including pediatric disclosure (disclosing children's HIV infection to children), parental disclosure (disclosing parental HIV infection to children), partner disclosure (disclosing HIV infection to partners), and social disclosure (disclosing HIV infection to the wider community beyond). Furthermore, the papers explore a number of factors within the process of HIV disclosure, including cognitive appraisal, privacy management, psychological well-being, health behaviors, and health ethics and patients’ rights. The global HIV epidemic affects widely different populations and, in this special issue, coverage of diverse groups of populations was deemed important to broaden the understanding. Thus, the studies include HIV-infected adolescents, HIV-infected parents, caregivers of HIV-infected children, HIV-infected adults, and HIV-infected men who have sex with men (MSM). In line with different demands, needs, and responses, the studies focused on different types of HIV disclosure among the different populations. Such understanding is key if a thorough understanding of the various outcomes that can be affected by HIV disclosure is to be built. Such outcomes include sexual behaviors, contraception use (Toska, Cluver, Hodes, Kidia, & Thabeng, 2015), physical, mental and social outcomes (Vreeman, Scanlon, Marete, Inui, & Nyandiko, 2015), treatment adherence (Qiao et al., 2015), and clinical outcomes (Itemba et al., 2015).

We include three studies focusing on issues related to HIV disclosure to children (either pediatric disclosure or parental disclosure) in resource-poor settings in Africa and Asia. Vreeman and colleagues investigated disclosure status among HIV-infected children aged 10–15 years in Western Kenya and its association with physical, mental, and social outcomes. The children who were aware of their HIV infection did not report worse psychosocial outcomes in terms of behavioral and emotional difficulties, depression, and quality of life. A number of factors were associated with a child's knowledge of his/her HIV diagnosis including older age (OR 1.8, 95% CI 1.5–2.1), better disease stage (OR 2.5, 95% CI 1.4–4.4), and fewer reported caregiver-level adherence barriers (OR 1.9, 95% CI 1.1–3.4; Vreeman et al., 2015). The second study was conducted among the caregivers in Ghana who had not disclosed children their HIV status, examining whether the characteristics of caregivers could predict their HIV-related knowledge, stigma, and discrimination, as well as illness perception (Paintsil et al., 2015). The study indicated that HIV-negative status and low level of formal education were significantly associated with poor HIV knowledge, and HIV-positive status was related to higher level of stigma perception (Paintsil et al., 2015). The third study focused on parental disclosure and was conducted with HIV-infected parents in China with children aged 5–16 years. The study showed that enacted (or experienced) stigma partially mediated the relationships between disclosure and mental health and, of specific importance, medicine adherence (Qiao et al., 2015).

Given the vulnerabilities and resiliencies associated with their unique developmental stages, HIV disclosure among adolescents living with HIV can be a particularly complex process. Special attention from the field to disclosure issues among this population was reflected in several studies in this collection. Nostlinger and colleagues explored social disclosure of HIV status (e.g., disclosure to individuals outside the family) by HIV-infected adolescents aged 13–17 years in Uganda and Western Kenya. They found that higher level of perceived self-efficacy to disclose, greater family support, less stigma, and higher self-esteem were associated with social disclosure (Nöstlinger, Bakeera-Kitaka, Buyze, & Buvé, 2015). Toska and colleagues studied HIV-infected adolescents aged 10–19 years in South Africa and examined how knowledge of HIV status by HIV-infected adolescents and their partners was associated with safer sexual activity and contraception use (Toska et al., 2015). However, neither knowing their partner's status nor disclosing one's HIV status to a partner was significantly associated with safer sex. These findings challenge assumptions that disclosure is automatically protective in sexual and romantic relationships for HIV-positive adolescents. Qualitative data further indicated that the fear of rejection, stigma, and public exposure after disclosing to partners was the main barrier to disclosure, while counseling by healthcare workers for HIV-positive adolescents did not address these fears and risks (Toska et al., 2015).

Several studies explored the practices and perceptions on HIV disclosure, especially partner disclosure, among adult populations. Itemba and colleagues assessed demographic correlates of HIV disclosure in a cohort of HIV-infected adults in Northern Tanzania. They reported that married individuals, those with secondary education, and those diagnosed earlier were more likely to have disclosed their HIV status to larger numbers of friends and relatives (Itemba et al., 2015). Another study collected qualitative data among women living with HIV in a rural region with a high HIV prevalence. The study found that being in a long-term and stable partnership is a main facilitator of partner disclosure, and trust, love, loyalty, and support were intertwined with women's sexual choices. Disclosure did not necessarily facilitate low-risk behaviors, and discordant couples may need support and counseling services that go beyond just the disclosure issues (Mkwanazi, Rochat, Tomlinson, Stein, & Bland, 2015).

Xiao et al. (2015) employed a communication theory perspective to examine reasons for disclosing or not disclosing HIV status to sexual partners among HIV-infected adults in China. The “communication privacy management” theory (Petronio, 2000) was used as the conceptual framework to investigate if key beliefs regarding disclosure were related to the HIV-infected adults’ decision-making of partner disclosure. Fear of rejection and concerns about privacy were significant barriers to partner disclosure, while endorsement of duty to inform/educate and motivation to establish a close/supportive relationship might promote disclosure (Xiao et al., 2015).

Walker and colleagues addressed ethical issues related to HIV disclosure in the Chinese socio-cultural context, with a focus on exploring how to respect patients’ autonomy, rights, and privacy. The paper highlighted that the concept of patient rights is compatible with Chinese culture. With respect for patients’ rights and autonomy, Chinese cultural practice of engaging families in care could be promoted in HIV care. In addition, healthcare providers have a duty to disclose truthfully the diagnosis and prognosis to HIV patients, and should receive training in disclosure ethics and skills (Walker, Nie, Qiao, Tucker, & Li, 2015).

Murphy and de Wit investigated disclosure expectations and practices among HIV-negative or status-unknown MSM in Australia through an on-line national self-report survey. Over three quarters of the participants expected HIV-positive partners to disclose their HIV status before sexual encounters, and 42% also expected HIV-negative men to declaim their HIV status although less than half consistently discussed their HIV status with sexual partners. Multivariate linear regression analysis suggested that men who expected HIV-positive partners to disclose their HIV status before sex more often lived outside capital cities, were less educated, were less likely to self-identify as gay, perceived more risk of HIV transmission via a range of sexual practices, were less engaged with the HIV-positive community, and expressed more stigma towards people living with HIV (Murphy & De Wit, 2015).

The studies in this special issue represent some of the most recent developments in research on HIV disclosure. At the same time, they collectively also reveal current knowledge gaps and future needs. To better understand the dynamics of decision-making disclosure and the actual disclosure process, we need to develop more complex theoretical models to account for multiple disclosures and the evolving ramifications of disclosure in emotional, behavioral, and legal domains. In terms of methodology, the field needs more longitudinal data, more comprehensive measures including psychosocial behavioral scales and biomarkers. In the practice of HIV prevention and care, it is important to develop or adapt evidence-based behavioral interventions to assist HIV-infected people to perform appropriate disclosure, so the benefits of disclosure to them and their family can be maximized (Kennedy, Fonner, Armstrong, O'Reilly, & Sweat, 2015; Siberry & Allison, 2015). All the efforts in the three aspects will contribute to a higher level of insights which in time will ameliorate the burden of secrecy, facilitate the process of disclosure, and enhance the well-being and adjustment of those challenged with disclosure needs.
